# Integrated Approach for Species Identification and Quality Analysis for *Labisia pumila* Using DNA Barcoding and HPLC

**DOI:** 10.3390/plants10040717

**Published:** 2021-04-07

**Authors:** Auni Aqilah Ahmad Tarmizi, Alina Wagiran, Faezah Mohd Salleh, Lee Suan Chua, Farah Izana Abdullah, Rosnani Hasham, Suraiya Binte Mostafiz

**Affiliations:** 1 Department of Biosciences, Faculty of Science, Universiti Teknologi Malaysia, Johor Bahru 81310, Malaysia; aunitarmizi@gmail.com (A.A.A.T.); faezah@utm.my (F.M.S.); suraiya.mouley@gmail.com (S.B.M.); 2 Institute of Bioproduct Development, Universiti Teknologi Malaysia, Johor Bahru 81310, Malaysia; lschua@ibd.utm.my (L.S.C.); farah.izana@utm.my (F.I.A.); r-rosnani@utm.my (R.H.); 3 Department of Bioprocess and Polymer Engineering, School of Chemical and Energy Engineering, Faculty of Engineering, Universiti Teknologi Malaysia, Johor Bahru 81310, Malaysia

**Keywords:** *Labisia pumila*, DNA barcoding, *ITS2*, *rbc*L, herbal medicinal products (HMPs), HPLC, authentication

## Abstract

*Labisia pumila* is a precious herb in Southeast Asia that is traditionally used as a health supplement and has been extensively commercialized due to its claimed therapeutic properties in boosting a healthy female reproductive system. Indigenous people used these plants by boiling the leaves; however, in recent years it has been marketed as powdered or capsuled products. Accordingly, accuracy in determination of the authenticity of these modern herbal products has faced great challenges. Lack of authenticity is a public health risk because incorrectly used herbal species can cause adverse effects. Hence, any measures that may aid product authentication would be beneficial. Given the widespread use of *Labisia* herbal products, the current study focuses on authenticity testing via an integral approach of DNA barcoding and qualitative analysis using HPLC. This study successfully generated DNA reference barcodes (*ITS2* and *rbc*L) for *L. pumila* var. *alata* and *pumila*. The DNA barcode that was generated was then used to identify species of *Labisia pumila* in herbal medicinal products, while HPLC was utilized to determine their quality. The findings through the synergistic approach (DNA barcode and HPLC) implemented in this study indicate the importance of both methods in providing the strong evidence required for the identification of true species and to examine the authenticity of such herbal medicinal products.

## 1. Introduction

Herbal medicine consists of herbs, herbal preparations, and mixtures of finished products that contain active ingredients from plant parts or plant materials [1]. In many developed countries, the World Health Organization (WHO) estimates that about 70–80% of the population has used a form of alternative or complementary medicine [1,2]. In Malaysia, many local food supplements and herbal medicines including *Labisia pumila*-based plants have been commercialized on the market, as the demand has grown over time. As the herbal industry continues to experience growth, quality control must be done. The monograph has been published as a referral standardized operating document for the sourcing of raw material, analytical testing techniques, and safety information [3]. Herbal medicinal products come in various forms, commonly in processed or modified forms (dried material, tablets, powders, capsules, or tablets), thereby presenting a challenge in accurately distinguishing genuine products from fake ones [4]. Consumers often rely on labels of product packages to be informed of their content; however, due to unscrupulous activities, manufacturers may fail to reach the presented quality and efficacy during production. Therefore, the correct identification of such species and their authenticity is vital to ensure their quality and to make them safer for consumption. Intensive tissue culture propagations of *Labisia pumila* as a high-quality raw product material have been performed [5,6]; however, the market demand exceeds the supply. Several factors contribute to a dwindling of supply such as uncontrolled harvesting activities from the wild, limited cultivation area, and high dependency on imported raw materials. This situation eventually led to the adulteration of raw materials, thus generating poor-quality products. The issue of quality is worsened if no efficient authentication tools are available to monitor the raw material quality and outcome of manufacturing procedures. Hence, the medicinal efficacy of the product may be jeopardized upon entering the market. 

The HMPs from *L. pumila* reportedly benefit consumers in terms of general health maintenance, and they have been used for inner health treatment, easing childbirth, and estrogen replacement therapy for women, as well as other potential treatments for chronic diseases [7,8,9,10,11,12,13] According to the Malaysia National Pharmaceutical Regulatory Agency (2020), most registered *Labisia* HMPs on the Malaysian market are in the form of capsules (84%), teabags (4%), tablets (3%), emulsions (1%), and bolus (1%), among others. *Labisia pumila* (family Myrsinaceace) commonly known as Kacip Fatimah (KF) is one of the most famous herbal plants which has a variety of local names [4]. Among the common three varieties, only *alata* and *pumila* are the commonly used terms in traditional medicine preparation [7,12,14]. Even though not much information can be found regarding the safety of *Labisia* products, consumption of *Labisia* water extract has resulted in unpleasant experiences due to assumedly adverse events [15,16]. Even though no serious adverse effects of *Labisia* herbal medicinal products for consumption have been reported so far, development of authentication methods is crucial to ensure their safety and efficacy. Various Malaysian government bodies have worked together to develop comprehensive monographs containing specifications for profiling techniques for raw material sourcing until approval [3]. Additionally, the Forest Research Institute Malaysia (FRIM) laboratory provides testing services and acts as an advisor to HMP manufacturers [17]. 

Chemical fingerprinting techniques such as HPLC have commonly been used for the determination of chemical markers in *L. pumila* plants. However, tandem LCMS/MS was reported recently for the determination and identification of HMPs of *L. pumila* var. *alata* using quercetin and myricetin [18]. A combination of HPLC and infrared (IR) spectroscopy methods was implemented to detect marker compounds in *L. pumila* plants and HMPs [19]. Several bioactive constituent of *L. pumila* have been identified, mostly by HPLC in the extract, including flavonoids (rutin, apigenin, kaempferol, naringin, and myricetin) and phenolic compound (gallic acid, caffeic acid, and pyrogallol) [8,20,21,22,23]. Despite the high demand for *Labisia* product on the market, the efficacy and safety of *L*. *pumila* HMPs in long-term usage is highly unclear, since very limited clinical data have been reported in animal studies [24,25]. Usage of chemical fingerprinting has also shown several drawbacks, such as the lack of a specific chemical marker for each herbal species, while mixtures of different plant species in herbal medicine products may gave different results due to different chemical compounds. The different batches of raw material sources used might affect their quality due to geographical location, storage conditions, or processing methods [26], posing difficulties for proper chemical analyses and objective judgment [27], which contribute to unreliable data. In addition, mixtures of several plant species may exhibit different chemical markers, and they may not be species-specific. Safety issues related to HMPs have received considerable attention according to several reports [28,29,30]; thus, effective detection methods are urgently required for more comprehensive identification. 

The foundation of an authenticity method based on DNA has been globally used since the development of DNA barcoding, a powerful tool in molecular biology [31,32]. These techniques have been used until now for the detection of various families of medicinal plants, as well as herbal medicine product authentication [33,34]. The DNA barcode was simplified as a unique identifier, specifically for each plant species. These methods can accurately discriminate the same species with similar morphological and chemical compounds, as well as exhibit repeatability. In the last 10 years, DNA barcoding methods have been rapidly applied worldwide for the identification of various species; however, the lack of standard reference material (SRM) may have hindered the progress of DNA barcoding, including for *L. pumila.* Therefore, the present study was undertaken to investigate the potential of DNA barcoding for *L. pumila* varieties, with the hope of enriching the GenBank database. Then, these DNA barcodes (*ITS2* and *rbc*L) were used for the identification of *L. pumila* in HMPs, while HPLC were performed for their quality assessment. Therefore, this study comprehensively applied dual methods for authentication to reveal the extent of adulteration existing in these herbal medicine products.

## 2. Results

### 2.1. L. pumila DNA Reference Barcode Generation

**Figure 1:**
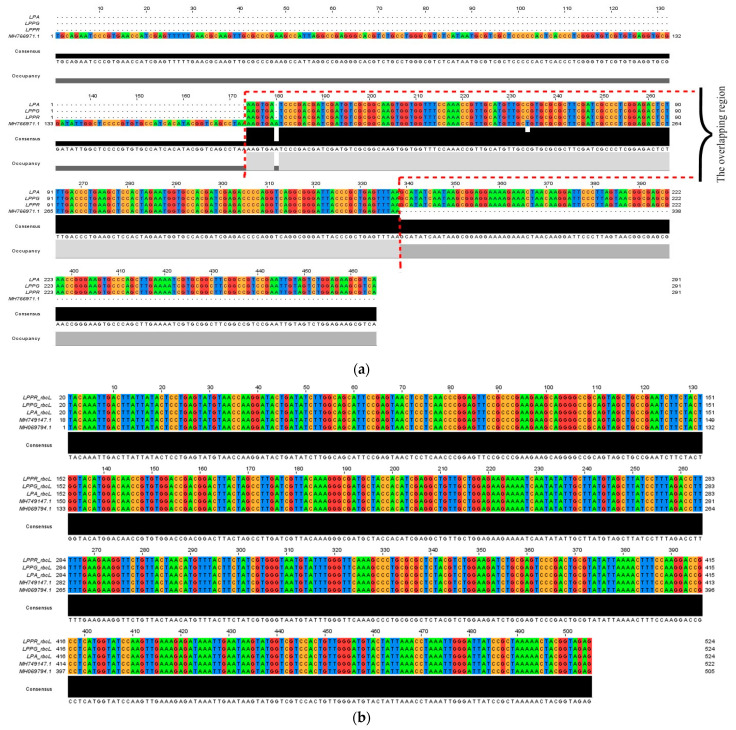
(**a**) Multiple sequence alignment of *ITS2* gene (291 bp) from *Labisia pumila* leaves (var. *pumila* green leaf (LPPG), var. *pumila* red leaf (LPPR), and var. *alata* (LPA)) with *L. pumila* var. *lanceolata* (MH766971.1) (338 bp) aligned using Jalview software. The conserved region shows nucleotides overlapping at base 175 until 339 (164 bp). The nucleotides are coded according to color; A: adenine (green), T: thymine (blue), G: guanine (red), C: cytosine (yellow). (**b**) Multiple sequence alignment of *rbc*L gene (523 bp) from *L. pumila* leaves (LPPG_*rbc*L, LPPR_*rbc*L, and LPA_*rbc*L) with *rbc*L sequences of *L. pumila* var. *lanceolata* (MH749147.1) and *L. pumila* (MH069794.1) previously generated by Tnah et al., 2019 [35] and Zuniga et al., 2017 [23] aligned using Jalview software. The nucleotides are coded according to color; A: adenine (green), T: thymine (blue), G: guanine (red), C: cytosine (yellow).

In the present study, the sequencing result of the amplified *ITS2* revealed that the length of the generated *L. pumila ITS2* was 291 bp. The analyzed sequences of the barcode region also revealed that the *ITS2* barcode region showed a high degree of identity, which was 98.79% similar to the GenBank hit generated [35] of *L. pumila* var. *lanceolata* (MH766971.1) from BLASTn (Basic Local Alignment Search Tool analysis (Figure 1). This was the only comparable *ITS2* reference sequence data from *L. pumila* plant that was previously deposited in the GenBank database. Since the size of the *ITS2* barcode of *L. pumila* var. *lanceolata* (MH766971.1) was bigger (338 bp) than the *L. pumila ITS2* barcode (291 bp) generated in this study, the query coverage between sequences was much lower (56%) due to the position of overlapping regions of *ITS2* barcodes.

In this study, the sequencing result of the amplified *rbc*L revealed that the length generated was 523 bp. The analyzed sequences of the barcode region also revealed that the *rbc*L showed a high degree of identity with other plants of the *L. pumila* species but differences in varieties after BLASTn with GenBank (Figure 1). The BLASTn result revealed that the *L. pumila rbc*L barcode regions generated from this study were 100% similar to *rbc*L sequences of *L. pumila* var. *lanceolata* (MH749147.1) and *L. pumila* (MH069794.1) previously generated [35]. All the sequences of *rbc*L and *ITS2* barcodes were successfully deposited into the GenBank database with the following references: MK249864.1 (LPPG), MK249865.1 (LPPR), and MK249863.1 (LPA); MH838010.1 (LPPG), MH838008.1 (LPPR), and MH828448.1 (LPA) respectively. 

### 2.2. Species Identification of L. pumila HMPs via DNA Barcoding

In this study, amplification of DNA barcodes was successfully performed using only six products (6/15). According to DNA barcoding sequence analysis, 20% of HMPs were considered authentic, while 13% of them were substituted, and the remaining 7% were considered contaminated. The other nine samples representing 60% (Kacip Fatimah product 4 (KFP4), KFP5, KFP6, KFP7, KFP8, KFP9, KFP10, KFP14, and KFP15) were not amplifiable due to the absence or low concentration of DNA recovery caused by degradation. These samples were declared as “no sequence” (NS), as described in Table 1. The sequencing analysis showed that the *ITS2* sequences generated for the *L. pumila* HMPs ranged from 290–339 bp, while those for *rbc*L the sequences generated were in the range of 523–552 bp, comparable to the sequence length deposited by [35,36]. The BLASTn results of the *rbc*L barcode region revealed that three of the tested HMPs (KFP1, KFP11, and KFP13) showed 100% BLAST search hits with the correct species of *L. pumila* var. *pumila* (MK249865.1) (Table 1). However, KFP2 showed the highest identity to *Nigella arvensis* (MG946921.1), while both KFP3 and KFP12 matched to *Camellia oleifera* (MF541730.2) with 99% to 100% similarities, respectively.

The BLASTn results of the *ITS2* barcode region revealed that, among all six of the *L. pumila* HMPs, four samples (KFP1, KFP11, KFP12, and KFP13) had 100% similarities to *L. pumila* var. *pumila* (MH838010.1). The other HMPs (KFP2 and KFP3) were identified as having been substituted with other plants species (*Anethum graveolens* and *Cuminum cyminum*). On the contrary, use of the *rbc*L barcode in KFP12 showed contamination because of presence other species not declared on the packaging label. The *rbc*L barcode showed 100% similarity to *Camellia oleifera* (MH838010.1). However, the correct species, *Labisia pumila* was detected when using the *ITS2* barcode. Substitution was detected in KFP2 and KFP3 after BLASTn analysis using *ITS2* and *rbc*L. The use of *rbc*L and *ITS2* DNA barcodes showed 99% and 98% similarity to two other species: *Anethum graveolens* (KM210329.1) and *Nigella arvensis* (accession number MG946921.1) respectively. The KFP3 teabag product was found to contain different plant species (*Cuminum cyminum*; accession number KX108698) when using *ITS2* as the DNA barcode. Application of the *rbc*L barcode revealed the nonmatching species intended for KFA3. Failure in the amplification of DNA barcodes from other samples posed a challenging issue due to insufficient genomic DNA (gDNA) recovered from degraded samples of HMPs. 

### 2.3. HPLC Fingerprinting of the L. pumila HMPs 

In this work, six authentic HMPs from DNA barcoding studies were further analyzed quantitatively for the presence gallic acid and rutin. Gallic acid is a bioactive marker compound that is abundantly found in *L. pumila* [35,36,37]. In order to confirm that the analytical method employed was reliable, it was validated for linearity using calibration curves. The calibration curve was linear over the concentration range of 2–400 mg/L for gallic acid producing a linear regression equation of *y* = 76,966*x* − 18,004, where *x* is the concentration of gallic acid (mg/L) and *y* is the corresponding peak area. Meanwhile, the calibration curve of rutin was linear within the equation *y* = 33,615*x* − 11,476 over a concentration range of 0.2–10 mg/L. The correlation coefficients (*r^2^*) for gallic acid (Figure S1) and rutin (Figure S2) were found to be 0.9993 and 0.9843, respectively.

**Figure 2:**
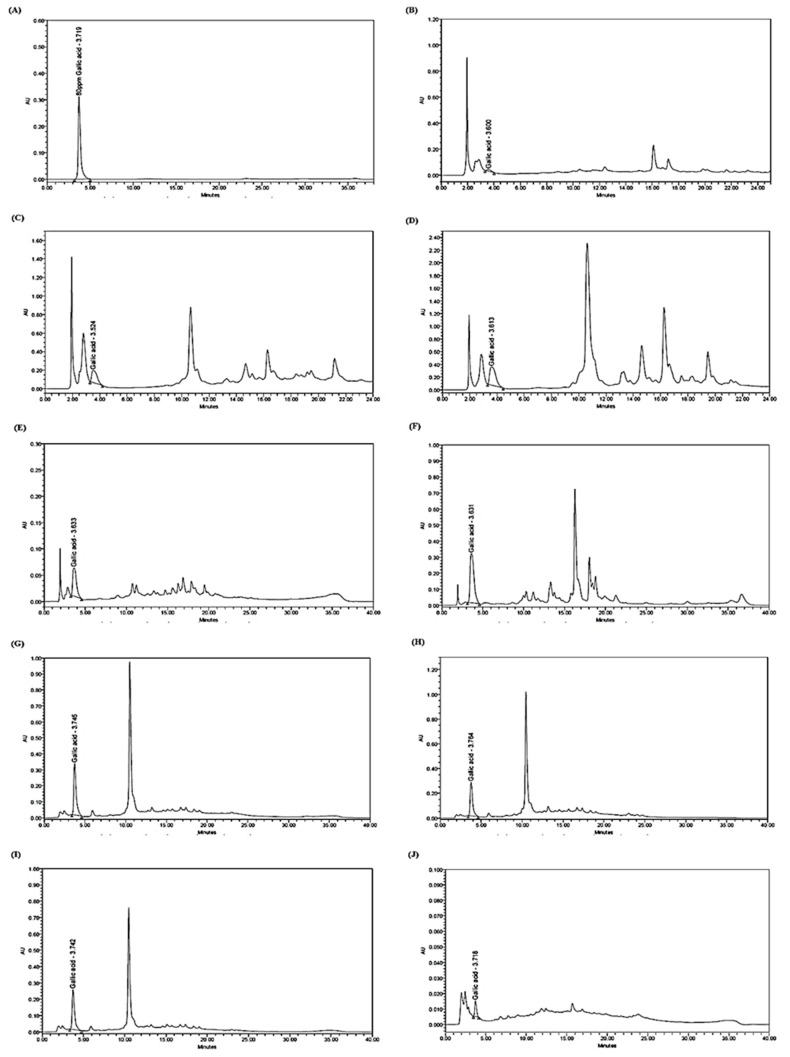
HPLC chromatograms of (**A**) gallic acid standard marker, (**B**) *L. pumila* var. *pumila* plant green leaf (LPPG), (**C**) *L. pumila* var. *pumila* plant red leaf (LPPR), (**D**) *L. pumila* var. *alata* plant (LPA), and *L. pumila* HMPs (**E**) KFP1, (**F**) KFP2, (**G**) KFP3, (**H**) KFP11, (**I**) KFP12, and (**J**) KFP13.

The detection of gallic acid and rutin in *L. pumila* HMPs was carried out according to the retention time of the standard and the *L. pumila* plant extracts. The spectral peaks of gallic acid and rutin were at 280 and 357 nm, respectively. The total run time for gallic acid detection was 40 min, and the peak was detected at a retention time of 3.719 min, similar to that of the three *L. pumila* leaf extracts and all six tested HMPs (KFP1, KFP2, KFP3, KFP11, KFP12, and KFP13). The peak patterns of *L. pumila* var. *alata* (LPA) compared to *L. pumila* var. *pumila* (LPPG), and *L. pumila* var. *pumila* (LPPR) as seen in Figure 2B–D showed a distinct single peak of gallic acid compound comparable to the gallic acid standard in Figure 1A. However, as seen in Figure 2B, *L. pumila* var. *alata* (LPA) showed a smaller peak, as detected, and it was shown with a very low concentration of 3.58 mg/L of gallic acid quantified from the analysis. 

**Figure 3:**
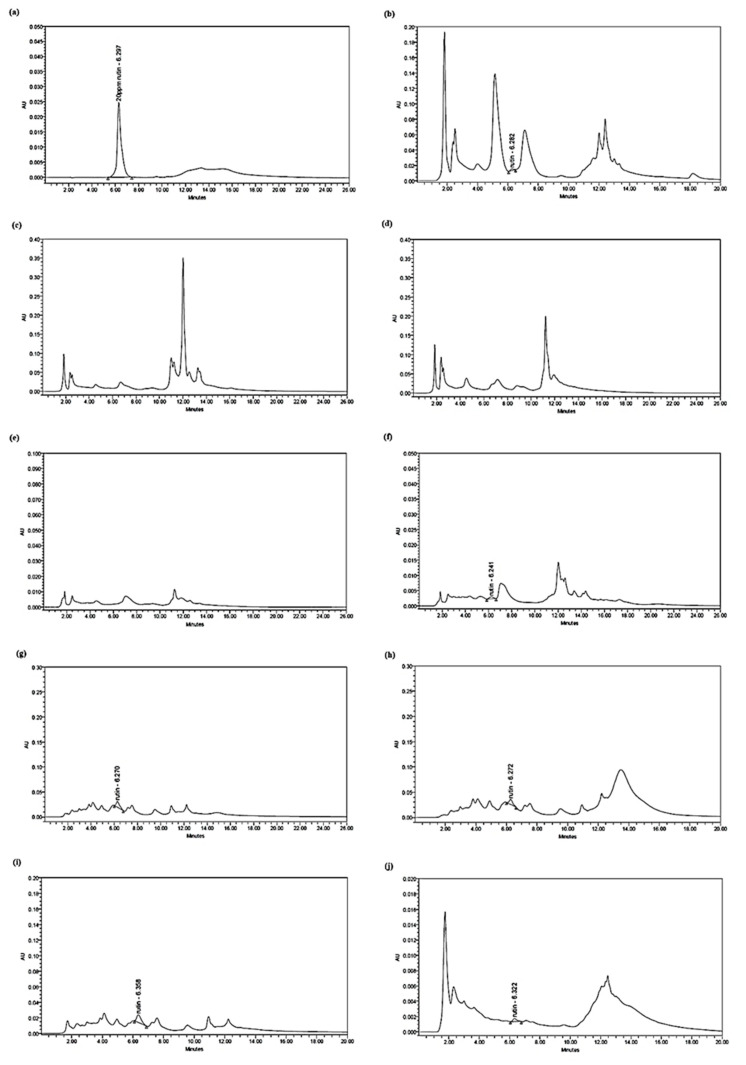
HPLC chromatograms of (**a**) rutin standard marker, (**b**) *L. pumila* var. *pumila* plant green leaf (LPPG), (**c**) *L. pumila* var. *pumila* plant red leaf (LPPR), (**d**) *L. pumila* var. *alata* plant (LPA), and *L. pumila* HMPs [(**e**) KFP1, (**f**) KFP2, (**g**) KFP3, (**h**) KFP11, (**i**) KFP12, and (**j**) KFP13].

The HPLC chromatograms in Figure 2 show that all six tested HMPs samples (100%), regardless of whether they were considered authentic, substituted (KFP2 and KFP3), or contaminated (KFP12) on the basis of DNA barcoding, contained the expected gallic acid chemical marker at the expected retention time when compared to the gallic acid standard in Figure 2A. Gallic acid as detected in substituted HMPs (KFP2 and KFP3) proving that the compound is not unique to *L. pumila* and might also be present in other species such as *Anethum graveolens*, *Nigella arvensis, Cuminum cyminum*, and *Camellia oleifera*. These other species were identified using the DNA barcoding method (Table 1). In the present study, detection of another compound, rutin, was employed to improve the detection of bioactive compounds. A total run time of 26 min was conducted for rutin detection, and the compound peak was detected at a retention time of 6.297 min. The results of the chromatogram in Figure 3 display only *L. pumila* var. *alata* (LPA), whereby five of the tested HMPs (KFP2, KFP3, KFP11, KFP12, and KFP13) had a rutin peak. Short and small separated peaks were also observed in the samples with rutin detection, indicating that the concentration was very low. The HPLC analysis implies that the contents of gallic acid and rutin, as seen in Table 2, varied between the samples. The concentration of gallic acid was highest in the red leaf extract of *L. pumila* var. *pumila* (LPPR) at a concentration of 113.93 mg/L, followed by green leaves of *L. pumila* var. *pumila* (LPPG) at 47.10 mg/L, and lowest for *L. pumila* var. *alata* (LPA) at 3.58 mg/L. Meanwhile, the concentration of gallic acid in all six tested HMP (KFP1, KFP2, KFP3, KFP11, KFP12, and KFP13) samples ranged from 2.23–132.17 mg/L. Low concentrations of rutin were detected for *L. pumila* var. *alata* (LPA) at 1.26 mg/L, whereas, for the HMPs (KFP2, KFP3, KFP11, KFP12, and KFP13), the concentrations ranged from 0.56–7.40 mg/L.

## 3. Discussion

### 3.1. The Establishment of L. pumila DNA Barcodes and HMP Authentication

**Figure 4:**
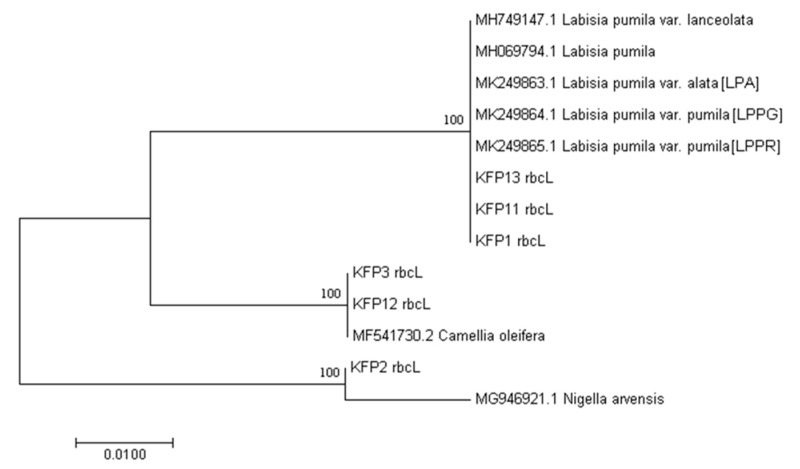
Neighbor-joining (NJ) tree of *L. pumila* HMP *rbc*L trimmed sequences compared to reference sequences generated in this work (MK249863.1, MK249864.1, and MK249865.1) and other DNA reference barcodes retrieved from GenBank (MH749147.1 and MH069794.1). KFP: Kacip Fatimah product. Numbers on the nodes of each branch are the bootstrap values for 1000 replicates.

**Figure 5:**
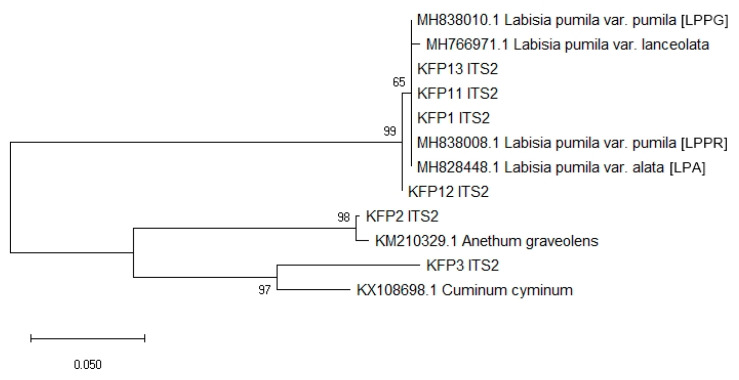
NJ tree of *L. pumila* HMPs *ITS2* trimmed sequences compared to reference sequences generated in this work (MH828448.1, MH838008.1, and MH838010.1) and another DNA reference barcode retrieved from GenBank (MH766971.1). KFP: Kacip Fatimah product. Numbers on the node of each branch are the bootstrap values for 1000 replicates.

Considering the morphological distinctness between the two varieties of *L. pumila* (var. *alata* and var. *pumila*) sequence analyzed in this work, the present study contributes to the GenBank data. Novel high-quality bidirectional barcodes for the two varieties of *Labisia* var. *alata* (LPA) and var. *pumila* (LPPG, LPPR) were successfully amplified using *rbc*L (523bp) and *ITS2* sequences (291bp). The present study proves the enrichment of GenBank databases with the three additional sequences of *L. pumila* for *ITS2* and another three for *rbc*L. The studies revealed a very low success rate of amplification (40%) regardless of DNA barcodes applied for HMPs. According to Table 1, amplifiable barcodes were from KFP1, KFP2, KFP3, KFP11, KFP12, and KFP13(40%), while 60% could not be barcoded even after several amplification attempts. Thus, this failure might be due to the type of HMP (capsule), which may contain high amounts of secondary metabolites from different varieties with additional formulation content such as excipients (fillers, lubricants, pigments, diluents, glidants, stabilizers, and binders) [10,17,33]. Previous reports also stated that successful DNA amplification from botanical materials (teas and roots) was higher compared to pharmaceutical formulations capsules, caplets, and tablets [38]. According to the phylogenetic neighbor-joining (NJ) analysis of *rbc*L HMPs in Figure 4, three amplified barcodes (KFP1, KFP11, and KFP13) formed a strongly supported monophyletic clade of bootstrap (BS) = 100 with *L. pumila* reference sequences generated in this work (MK249865.1, MK249864.1, MK249863.1) and other hits of similar species but different variety from GenBank (MH069794.1 and MH749147.1). In addition, NJ analysis of the *ITS2* barcode region in Figure 5 also revealed that four of the tested *L. pumila* HMPs (KFP1, KFP11, KFP12, and KFP13) formed a strongly supported monophyletic clade (BS = 99) with *L. pumila* reference sequences (MH838008.1, MH828448.1, and MH838010.1) established from this work and other reference sequences retrieved from the GenBank database, i.e., *L. pumila* var. *lanceolata* (MH766971.1). The results obtained from BLASTn analysis supported by NJ analysis indicated that three of the tested HMPs were truly authentic. Results showed that the other two *L. pumila* HMPs (KFP2 and KFP3) nested outside the *L. pumila* clade and clustered with *Anethum graveolens* for KFP2 and *Cuminum cyminum* for KFP3, suggesting substitution.

Sequence analysis of the *ITS2* DNA barcode from KFP2 was revealed to contain *Anethum graveolens* and *Nigella arvensis* instead of *L. pumila*, confirming substitution. *Anethum graveolens* is usually used as a spice, while *Nigella arvensis* is known as wild fennel [39,40]. The content of KFP3 is believed to have been substituted with spices *Cuminum cyminum* and *Camellia oleifera* when *ITS2* and *rbc*L were used, respectively. Undeclared plant species (*Camellia oleifera*) were detected, indicating contamination for KFP12. These results show that adulteration and ingredient substitution apparently occurred due to the addition of inferior species, suggesting fraudulent activities by manufacturers. In terms of efficiency, both *rbc*L and *ITS2* barcodes were equally ideal markers for the authentication of *L. pumila* HMPs. Using BLASTn and NJ tree criteria, a certain extent of authenticity and fraudulence was detected for *L. pumila* HMPs samples.

### 3.2. HPLC Fingerprinting of L. pumila HMPs 

The presence of a chemical marker is not sufficient to validate herbal medicine preparation; however, a minimal concentration of the chemical marker must be present [41]. In order to validate the first screening step for HMP authentication using DNA barcoding, further HPLC analysis was employed to detect gallic acid and rutin in HMPs. Gallic acid (GA) is a chemical constituent that primarily acts as an antioxidant and is found abundantly in all three *L. pumila* varieties. Previously, evidence on the antioxidant activities of gallic acid was restricted to results from in vitro experimentations with different human cell lines and blood cells [30,42]. However, further studies conducted on animals showed that it induces antioxidant enzymes and protects the liver against the effects of cytotoxicity [43,44,45]. On the other hand, rutin is a flavonol that also demonstrates several important pharmacological effects such as antioxidant, anticarcinogenic, vasoprotective, cytoprotective, neuroprotective, and cardioprotective activities. According to the results of the HPLC chromatogram shown in Figure 1, the gallic acid compound was detected at Rt 3.719 min and observed at a single peak in *L. pumila* var. *alata* (LPA), *L. pumila* var. *pumila* (LPPG), and *L. pumila* var. *pumila* (LPPR). However, additional peaks were also observed in the later elution, which might indicate the presence of other unidentified compounds such as aromatic and hydrophobic compounds. For commercialization purposes, *L. pumila* var. *pumila* and *L. pumila* var. *alata* are commonly used by manufacturers in HMP production [44]. Figure 1 shows that rutin was detected at Rt 6.297 min. Interestingly, it can be observed in Figure 1b that rutin was only detected in the *L. pumila* var. *alata* extract at Rt 6.282 min. In the case of *L. pumila* HMPs, only five of the tested samples (KFP2, KFP3, KFP11, KFP12, and KFP13) were found to contain rutin.

The HPLC analysis implied that the HMPs might comprise two or more plant species, and these plants might possess the same chemical marker detectable via chemical analysis without accurately identifying to which species they belong. The weakness of this might be misdiagnosis due to limitations and a lack of expertise. The results of HPLC may vary because of external factors such as temperature, humidity, and soil composition [24]. However, this does not corroborate the authenticity of these HMPs, as the presence of a bioactive compound marker may not indicate the presence of the label-declared plant species. The peaks observed in each chromatogram (Figure 1 and Figure 2) were also different for each *L. pumila* HMP (KFP1 was monoherbal, while the others were polyherbal). However, multiple peaks showed the possible presence of other compounds due to the complexity of the product content. According to Table 2, gallic acid concentrations were found highest in *L. pumila* var. *pumila* (LPPR) and KFP2 at 113.93 mg/L and 132.17 mg/L, respectively. Meanwhile, rutin was found at the highest level in the KFP3 sample at 7.40 mg/L. The table also shows that, for product KFP1, without detection of one of the tested bioactive compounds, this might lessen the product efficacy when being consumed. Previous work found that the threshold concentration for no adverse effects of gallic acid in rodents is 120 mg/kg, a dose equivalent to 2.9 g/day in a 70 kg man [45]. In the case of rutin, previous studies showed that data related to the compound’s potential toxicity support its safety as a dietary supplement with an intake range of 5 mg to 40 mg of rutin per day. Hence, the concentrations found in products were already lower than the suggested amounts to provide enough of a therapeutic effect for the consumer. Larger-scale analysis is required in the determination of a GA and rutin dosage standard, which should be extensively studied at the clinical level to establish an effective dosage standard for human consumption. High-added-value bioactive compounds are being actively extracted and used as HMP active ingredients, such as GA, which currently attracts substantial interest thanks to its powerful antioxidant properties. However, in the case of polyherbal preparations, the pharmacological effects of a certain compound might derive from all or some of the species listed. Since KFP2, KFP3, KFP11, KFP12, and KFP13 (Table 3) are known to contain various polyherbals listed in the ingredients list, it can be suggested that the detection of galic acid and rutin might not have solely been from the *L. pumila* plant. For instance, KFP2 is known to contain *Quercus infectoria* and *Parameria polyneura*. Detection of GA and rutin compounds for *Quercus infectoria* was reported; however, no data are available for *Parameria polyneura* [46]. KFP3 is known to contain *Pueraria mirifica* and *Camellia sinensis* (black tea leaves), in which gallic acid and rutin compounds have previously been detected [47,48]. 

The likelihood of KFP3 and KFP11 containing gallic acid and rutin compounds is higher due to *Camellia sinensis* (black tea leaves), while this is the case with *Pueraria mirifica* in KFP12. KFP13 also features a similar pattern due to *Chamaemelum nobile* that was reported to contain both gallic acid and rutin compounds [48]. The presence of other plant species in the products showed potential adulterations or substitutions in *L. pumila* HMPs despite the label claiming *L. pumila* as the active ingredient. The combination methods for *L. pumila* HMP authentication using DNA barcoding and HPLC analysis revealed that certain results were found to correlate with one another. This was shown in the three tested samples (KFP1, KFP11, and KFP13) identified as authentic using DNA barcoding and gallic acid measurements.

Usage of the dual methods of DNA barcoding and chemical analysis (HPLC) is, thus, beneficial in screening the authenticity and quality of HMPs. Previous reports showed that a combination of DNA barcoding and HPLC successfully identified five *Fritillariae bulbus* species from TCM (Traditional Chinese Medicine) and its effective chemical constituents [49]. The authenticity of *Eurycoma longifolia* HMPs has been tested by DNA barcoding using the *ITS2* marker, and its quality was verified using HPLC [50]. The quality of the famous *Curcuma longa* L. sold on the Chinese market was also assessed with DNA barcoding by discriminating the species using the *ITS/LSU D1/D3* marker and HPLC to determine the chemical compositions [51]. Previous reports successfully evaluated the quality and species of *Phellodendri cortex* using DNA barcoding with *ITS* and *psbA-trnH*, as well as HPLC fingerprinting [52,53]. The use of dual authentication methods (DNA barcoding and HPLC) for bigger datasets of 257 samples from the Brazilian market revealed a high level of substitution (71%) [41]. The present study showed that chemical methods cannot distinguish species that have similar chemical markers, possibly misidentifying the sources of raw materials, consistent with the findings from other researchers [3,52]. In summary, the present work showed the establishment of molecular analysis and chemical fingerprinting methods for discriminating *Labisia* herbal medicinal products. The results reveal that DNA barcoding overcomes the limitation of HPLC fingerprinting, potentiating its use for the accurate and scientific confirmation of herbal identities in medicinal materials from multiple sources. Nevertheless, the DNA barcoding approach presented here has a limiting factor in that it is unable to retrieve high-quality gDNA from several herbal medicinal products after several attempts, which are then deemed not amplifiable. The use of a normal PCR mixture for degraded DNA might not be suitable for severely degraded DNA. Therefore, we proposed a high-fidelity PCR mixture for severely degraded gDNA in the future. Once the high-quality gDNA is obtained, the use of a mini barcode (less than 200 bp) may be beneficial. There are no standardized chemical markers specifically for *L. pumila*; thus, the quality assessment of mixed HMPs might give misleading results. Calibration and development methods also take a longer time compared to DNA barcoding. 

## 4. Materials and Methods

### 4.1. Collection of Plant Samples and Herbal Medicinal Products

The *L. pumila* plants (var. *alata* and var. *pumila*) were collected from Agriculture Department, Parit Botak, Batu Pahat, Johor and sent to the Forest Research Institute Malaysia (FRIM), Kepong for species identification (Tables S1 and S2, Supplementary Materials). In this work, two morphologically different var. *pumila* with green and red leaves were verified as PID 250817-17 (LPPG) and PID 260817-17 (LPPR), respectively. *L. pumila* var. *alata* (LPA) was verified as PID 270817-17. In the present study, 15 HMPs (labeled as KFP) consisting of teabags and capsules were purchased from various retail shops in Johor, Malaysia and through an online website. 

### 4.2. Genomic DNA Extraction, PCR Amplification, and Sequencing 

Approximately 100 mg of fresh leaves and herbal medicinal products (HMPs) in different forms (approximately 150 mg) were extracted using a NucleoSpin Plant II Kit (Macherey-Nagel, Düren, Germany). The yield and purity of the gDNA extracted were analyzed using a spectrophotometric nanodrop with an absorbance ratio (A_260/280_). The quality of gDNA extracted from the plant and HMPs was examined on 1% (*w*/*v*) agarose gel electrophoresis. The high-quality genomic data were then used for PCR amplification. Amplification of the chloroplast barcode of ribulose-1,5-bisphosphate carboxylase/oxygenase large subunit (*rbc*L) and nuclear barcode internal transcribed spacer 2 (*ITS2*) genes were carried out on a MJ Mini™ Thermal Cycler from Bio-Rad (Bio-Rad Laboratories, Inc., Hercules, CA, USA). The gDNA template (50–100 ng/µL) was added to the PCR mixture containing 5 µL of 5× PCR buffer, 2 µL of MgCl_2_, 1 µL each of forward and reverse primers (10 mM), 0.4 µL of dNTP(deoxyribonucleotide triphosphate) mix, 0.625 U of Taq DNA Polymerase (Promega, Madison, WI, USA), and sterile distilled water. The primers used in the PCR amplification of both barcode regions were *rbc*L_F ATGTCACCACAAACAGAGACTAAAGC [54], *rbc*L_R GTAAAATCAAGTCCACCRCG (Kress and Erickson, 2007), and *ITS2*_F GGGGCGGATATTGGCCTCCCCTTGC, *ITS2*_R GACGCTTCTCCAGACTACAAT [28]. The PCR amplification profile for *rbc*L region was as follows: 95 °C for 2 min, followed by 30 cycles at 95 °C for 1 min, 57 °C for 1 min, 72 °C for 1 min, and a final elongation step at 72 °C for 5 min. Next, for *ITS2*, the amplification was programmed as follows: 95 °C for 2 min, followed by 30 cycles at 95 °C for 1 min, 57 °C for 15 s, 72 °C for 1 min, and a final elongation step at 72 °C for 5 min. The PCR products were examined on 2% (*w*/*v*) agarose gel electrophoresis. The negative control reaction with no gDNA template was included in all experiments to ensure that the PCR mixture was free from contamination. Successfully amplified barcodes were sent to Apical Scientific Sdn. Bhd. (Seri Kembangan, Selangor, Malaysia) for bidirectional sequencing using an ABI3730xl Genetic Analyzer (Applied Biosystem, Foster City, CA, USA).

### 4.3. DNA Sequencing Analysis

To generate standard references barcodes, DNA barcode regions were trimmed and edited using the BioEdit software (version 7.2.5). The generated sequences were then submitted to BLAST in the GenBank (https://blast.ncbi.nlm.nih.gov/Blast.cgi, accessed on 5 December 2020) for identification of a closely related species match. The sequences generated along with the selected closely matched sequences in the BLASTn search were downloaded in FASTA format and included in the analysis. All novel SRM sequences were subsequently deposited into National Center of Biotechnology Information (NCBI) database. The HMP authenticity was assessed by two criteria: 97% top match or above, and neighbor-joining (NJ) method based on the Kimura two-parameter model (K2P) evaluation using 1000 bootstrap (BS) replicates. The HMPs containing *Labisia* were considered “authentic” if a DNA barcode matched to the species. However, they were considered “substituted” if other species of DNA barcodes were found other than those labeled on the HMP with no barcode detected for the main ingredient. The HMP was considered “contaminated” if a DNA barcode was found other than that on the HMP packaging label, in addition to the authentic barcode. Lastly, the HMP was considered “no sequence” (NS) if the DNA was unable to be retrieved from the tested HMP [29,54].

### 4.4. Chemicals and Reagents for HPLC Analysis

To determine the presence and concentration of bioactive markers in the *L. pumila* leaves and HMP extracts, an HPLC chromatographic fingerprint analysis was performed. The stock concentration of reference standards gallic acid (Sigma-Aldrich) and rutin (Sigma-Aldrich) were separately prepared, and working standard solutions were produced before calibration testing. Gallic acid was further diluted to produce working standard solutions with concentrations in parts per million (ppm) of 400 ppm, 200 ppm, 100 ppm, 80 ppm, 20 ppm, and 2 ppm. Meanwhile, rutin was also prepared at concentrations of 10 ppm, 8 ppm, 5 ppm, 2 ppm and 0.2 ppm. The ppm unit is convertible to milligrams per liter (mg/L). All stock and working solutions were stored at 4 °C prior to HPLC analysis.

### 4.5. Preparation of L. pumila Leaves and HMP Extracts

The fresh leaves of *L. pumila* were oven-dried for 2 weeks at 45 °C and cut into very small pieces at a size of approximately 1 cm before preparation of the chromatographic analysis. Crude extracts were obtained from the leaves. Two grams of the dried *L. pumila* was accurately weighed and added into a reflux system containing 80 mL of boiled ethanol 70% (*v*/*v*) for 1 h. Using Whatman No. 1 filter paper, the infusion was then filtered and concentrated using a rotary evaporator (IKA® HRC 2 basic) at 50 °C. The concentrated filtrate was further dried in a freeze-drier (Christ) to form a powder. The *L. pumila* crude extract was stored at −20 °C in a freezer for subsequent analysis [55]. Approximately 250 mg *L. pumila* HMP capsules were accurately weighed and dissolved in 10 mL of HPLC-grade methanol. The methanol solution was vortexed and ultrasonically extracted for 15 min at room temperature in order to ensure the complete extraction of bioactive compounds. The sample solution was filtered through a 0.45 µm syringe filter (Sartorius) prior to HPLC analysis. For the teabag samples, the extraction was conducted as reported by [56,57,58]. Briefly, 250 mg of teabag contents were placed into a 100 mL conical flask containing 10 mL of ethanol 70% (*v*/*v*) which was brought to boiling point for 5 min. The sample solution was then allowed to cool before centrifuging for 10 min at 14,000 rpm at 1 °C. The supernatant was filtered through a 0.45 µm syringe filter (Sartorius) before HPLC injection.

### 4.6. Quantification of Gallic Acid and Rutin in L. pumila Leaves and HMP Extracts

HPLC analysis was performed at the Institute of Bioproduct Laboratory, UTM, using a Waters Alliance 2690 HPLC equipped with an ultraviolet (UV) detector [59]. Chromatographic separation was carried out using a Luna® C18 column (150 × 4.6 mm^2^, 5 µm). The chromatographic conditions were different for gallic acid and rutin. The mobile phase for gallic acid comprised acetonitrile and 3% phosphoric acid with a flow rate of 0.7 mL/min. Meanwhile, the conditions for rutin analysis comprised acetonitrile and 0.5% acetic acid at a similar flow rate of 0.7 ml/min. A volume of 20 µL of each sample was injected into the HPLC system, and the run times for gallic acid and rutin were maintained for 40 min and 26 min, respectively, throughout the analysis. The photodiode array detector (PDA) wavelengths were set at 280 nm for gallic acid and 357 nm for rutin. The linear regression equations obtained from gallic acid and rutin standard curves were applied to calculate the concentrations of both compounds in the *L. pumila* HMPs. This was also used to calculate the concentration of the standards in the plant extracts. The peak areas corresponding to gallic acid and rutin were assigned by comparing their retention times with those of the reference standard [60].

## 5. Conclusions

The present work contributes to the enrichment of reference barcodes for another two varieties of *L. pumila*. New molecular and chemical analysis methods were also established for distinguishing genuine HMPs containing *Labisia* spp. The results revealed that comprehensive and dual authentication methods strengthened the identification methods of *Labisia* species. The unique DNA barcodes generated in this study can be used for quality control and authentication of *Labisia* HMPs in the herbal industry.

## Figures and Tables

**Table 1:** Authentication summary of selected *L. pumila* herbal medicine products (HMPs) via DNA barcoding.

Code	Declared Ingredients	Barcode Region	BLASTn Best Hit	Identity (%)	Query Cover (%)	Accession No.	Barcode ID	Identity of Barcodes
KFP1 (capsule)	*Labisia pumila*	*ITS2*	*Labisia pumila* var. *pumila*	100	100	MH838010.1	*Labisia pumila* var. *pumila*	Authentic
		*rbc*L	*Labisia pumila* var. *pumila*	100	100	MK249865.1		
KFP2 (capsule)	*Labisia pumila, Quercus* *infectoria*, *Parameria* *polyneura*	*ITS2*	*Anethum graveolens*	99.29	89	KM210329.1	*Anethum graveolens, Nigella arvens*	Substituted
		*rbc*L	*Nigella arvensis*	98.65	100	MG946921.1		
KFP3 (capsule)	*Labisia pumila, Camellia sinensis*, *Pueraria mirifica*	*ITS2*	*Cuminum cyminum*	90.11	89	KX108698.1	*Cuminum cyminum, Camellia oleifera*	Substituted
		*rbc*L	*Camellia oleifera*	99.81	100	MF541730.2		
KFP4 (capsule)	*Labisia pumila*	NA	NS	NS	NS	NS	NS	NS
KFP5 (capsule)	*Labisia pumila, Querqus* *lusitanica*	NA	NS	NS	NS	NS	NS	NS
KFP6 (capsule)	*Labisia pumila, Quercus* *infectoria*	NA	NS	NS	NS	NS	NS	NS
KFP7 (capsule)	*Labisia pumila, Terminalia chebula*	NA	NS	NS	NS	NS	NS	NS
KFP8 (capsule)	*Labisia pumila, Croton* *caudatum*	NA	NS	NS	NS	NS	NS	NS
KFP9 (capsule)	*Labisia pumila, Nigella* *sativa, Goniothalamus macrophyllus, Elephantopus scaber, Terminalia chebula, Kaempferia galangal,* *Curcuma longa*	NA	NS	NS	NS	NS	NS	NS
KFP10 (capsule)	*Labisia pumila*	NA	NS	NS	NS	NS	NS	NS
KFP11 (tea bag)	*Labisia pumila, Camellia sinensis*	*ITS2*	*Labisia pumila* var. *pumila*	100	100	MH838010.1	*Labisia pumila* var. *pumila*	Authentic
		*rbc*L	*Labisia pumila* var. *pumila*	100	100	MK249865.1		
KFP12 (tea bag)	*Labisia pumila, Pueraria mirifica*	*ITS2*	*Labisia pumila* var. *pumila*	100	100	MH838010.1	*Labisia pumila* var. *pumila, Camellia oleifera*	Contamination
		*rbc*L	*Camellia oleifera*	100	100	MF541730.2		
KFP13 (tea bag)	*Labisia pumila, Chamaemelum nobile*	*ITS2*	*Labisia pumila* var. *pumila*	100	100	MH838010.1	*Labisia pumila* var. *pumila*	Authentic
		*rbc*L	*Labisia pumila* var. *pumila*	100	100	MK249865.1		
KFP14 (tea bag)	*Labisia pumila*	NA	NS	NS	NS	NS	NS	NS
KFP15 (capsule)	*Labisia pumila*	NA	NS	NS	NS	NS	NS	NS

NA: non-amplifiable; NS: no sequence; KFP: Kacip Fatimah product, BLAST: Basic Local Alignment Search Tool.

**Table 2:** Concentration (mg/L) of gallic acid and rutin in *Labisia pumila* leaf extract and HMPs.

Sample ID	Gallic Acid		Rutin	
	Retention Time (min)	Concentration (mg/L)	Retention Time (min)	Concentration (mg/L)
LPPG	3.524	47.10	ND	0.00
LPPR	3.613	113.93	ND	0.00
LPA	3.617	3.58	6.282	1.26
KFP1	3.633	21.94	ND	0.00
KFP2	3.631	132.17	6.241	0.79
KFP3	3.745	84.96	6.270	7.40
KFP11	3.764	73.45	6.272	4.94
KFP12	3.742	66.06	6.358	6.38
KFP13	3.718	2.23	6.322	0.56

ND: not detected.

**Table 3:** Summary of *L. pumila* HMP authentication using DNA barcoding and HPLC.

Serial Number	Product Type		Analysis	
		DNA Barcode	HPLC	
			Gallic Acid	Rutin
KFP1	Capsule	Authentic	Yes	No
KFP2	Capsule	Substituted	Yes	Yes
KFP3	Tea bag	Substituted	Yes	Yes
KFP11	Tea bag	Authentic	Yes	Yes
KFP12	Tea bag	Contaminated	Yes	Yes
KFP13	Tea bag	Authentic	Yes	Yes

## Supplementary Materials

The following are available online at https://www.mdpi.com/article/10.3390/plants10040717/s1, Table S1. The observable fresh and dried samples of *L. pumila* plants; Table S2. The observable contents of *L. pumila* HMPs; Figure S1. Calibration curve of gallic acid; Figure S2. Calibration curve of rutin.
