# Supplementary material for: Integrated Approach for Species Identification and Quality Analysis for Labisia pumila Using DNA Barcoding and HPLC
Source: Plants (Basel). 2021 Apr 7;10(4):717. doi: 10.3390/plants10040717 (PMC8067811; doi:10.3390/plants10040717)
Supplement: Supplementary file 1 [file plants-10-00717-s001.pdf]

Table S1. The observable fresh and dried sample of *L. punala* plants

Sample Type	Sample Id	Figure	
Labisia pumila var.*alata*	LPA (PID 270817-17)	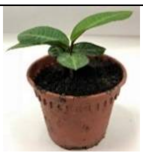	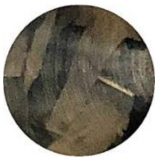
Labisia pumila var. *pumila* (Green leave)	LPPG (PID 250817-17)	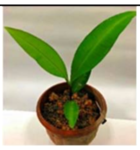	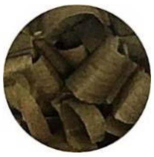
Labisia pumila var.*pumila* (Red Leave)	LPPR (PID260817-17)	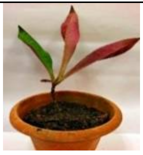	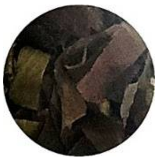

Table S2. The observable contents of *L. pumila* HMPs

Sample ID	Figure	Sample ID	Figure
KFP1	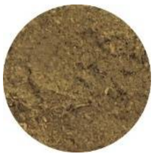	KFP11	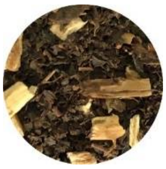
KFP2	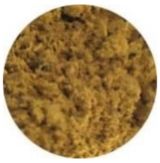	KFP12	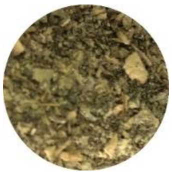
KFP3	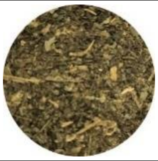	KFP13	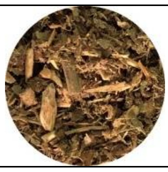

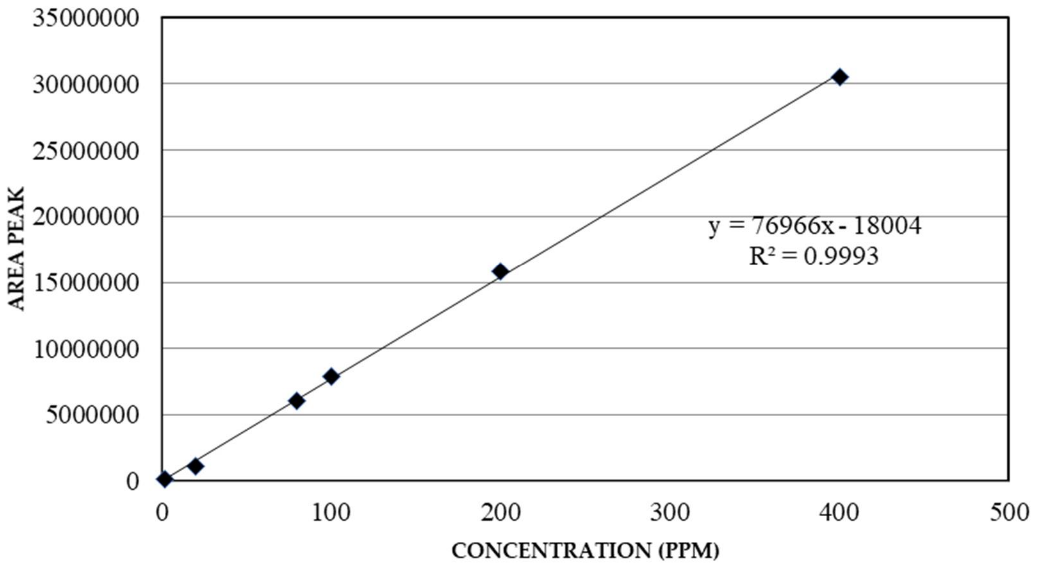

Figure S1: Calibration curve of gallic acid

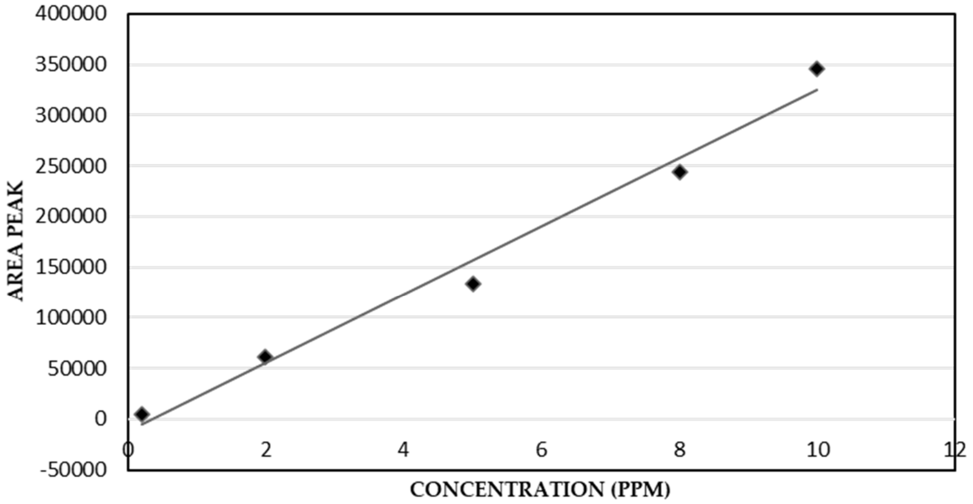

Figure S2: Calibration curve of rutin
